# Effect of slice thickness on quantitative analysis of interstitial lung disease: a retrospective volumetric chest CT study

**DOI:** 10.1007/s11547-025-02023-w

**Published:** 2025-05-27

**Authors:** Marijan Pušeljić, Borut Mohorko, Tadej Počivavšek, Florentine Moazedi-Fürst, Johannes Schmid, Michael Fuchsjäger, Emina Talakić

**Affiliations:** 1 Division of General Radiology, Department of Radiology, Medical University of Graz, Auenbruggerplatz 9, 8036 Graz, Austria; 2 Division of Rheumatology and Immunology, Department of Internal Medicine, Medical University of Graz, Graz, Austria

**Keywords:** Interstitial lung disease, Pulmonary fibrosis, Computed tomography, Quantitative lung analysis

## Abstract

**Introduction:**

High-resolution computed tomography (HRCT) is essential for evaluating interstitial lung disease (ILD). The effect of slice thickness on threshold-based quantification of individual ILD components remains underexplored. This study investigates the effect of slice thickness on ILD quantification using Lung CT Analyzer.

**Methods:**

Retrospective analysis of 53 ILD patients (mean age 64.3 ± 14.1 years) who underwent chest CT scans with HRCT (slice thickness ≤ 1.25 mm) and conventional CT (CCT, ≥ 2.5 mm) reconstructions. Quantitative lung volumes, functional parenchyma, emphysema, ground-glass opacity (GGO), consolidation and affected parenchyma were assessed. The effects of contrast media (CM) application and ILD pattern was assessed separately.

**Results:**

Emphysema volume was significantly higher in HRCT compared to CCT for the whole lung (766.9 ± 568.3 mL vs. 482.6 ± 454.4 mL, *p* < 0.001), the right lung (431.4 ± 314.6 mL vs. 270.2 ± 251.3 mL, *p* < 0.001), and the left lung (337.3 ± 259.9 mL vs. 228.0 ± 221.5 mL, *p* < 0.001). Consolidation volumes also differed significantly between HRCT and CCT for the whole lung (271.6 ± 128.4 mL vs. 252.0 ± 126.3 mL, *p* < 0.001), with similar findings for the right and left lung. Functional volume was underestimated in CCT reconstructions. No significant differences were observed for GGO volumes or overall affected parenchyma. CM application and ILD pattern had no significant interaction on the measurements.

**Conclusion:**

Slice thickness significantly affects the quantification of functional parenchyma, emphysema and consolidation, whereas GGO and the overall ILD extent remain unaffected.

**Supplementary Information:**

The online version contains supplementary material available at 10.1007/s11547-025-02023-w.

## Introduction

Interstitial lung disease (ILD) comprises a heterogeneous spectrum of disorders characterized by inflammation and fibrosis of the interstitial space, often progressing to pulmonary fibrosis and respiratory failure [1, 2]. The prevalence of ILD varies significantly across regions, with idiopathic pulmonary fibrosis, ILD associated with connective tissue disorders, and hypersensitivity pneumonitis being the most common subtypes [2–4]. High-resolution computed tomography (HRCT) is the recommended gold-standard imaging method for screening and monitoring patients with known or suspected ILD [5, 6]. However, precisely evaluating the complex morphology and extent of ILD remains challenging, as visual assessments often suffer from poor reproducibility and high interobserver variability [7, 8]. To address these limitations, quantitative lung analysis of chest CT scans has been extensively studied in the recent years [9]. Several computerized tools for lung segmentation and parenchymal analysis have been developed [9, 10], providing objective and reproducible results [11]. Among these, threshold-based quantitative analysis, which relies on pixel counts above or below defined attenuation thresholds, is frequently employed in this patient group to estimate the relative or absolute volumes of different parenchymal components [10].

Prior studies have predominantly explored how slice thickness affect the quantification of lung nodule [12–16] and emphysema volume [17–19]. Additionally, previous research has highlighted the influence of different reconstruction algorithms, such as high-frequency or smooth reconstruction kernels, on CT attenuation values, which can impact the results of quantitative lung analysis [20–23]. Notably, a study by Nguyen-Kim et al. [24] found that reducing slice thickness in HRCT leads to a higher lung density compared to scans with thicker slices. However, no previous studies have specifically evaluated the impact of slice thickness on threshold-based quantitative analysis of chest CT scans in patients with ILD, particularly with regard to individual parenchymal components.

The aim of this study was to evaluate the impact of varying slice thickness reconstructions on threshold-based quantitative volumetric analysis of ILD using the Lung CT Analyzer. We hypothesize that slice thickness will have distinct effects on high and low attenuation parenchymal changes as well as overall lung volume. Additionally, the influence of contrast media (CM) application and ILD pattern on the results will be assessed separately.

## Methods

### Study design

This retrospective, single-centre study received approval from our institutional ethics committee and adhered to the principles of the Declaration of Helsinki. The requirement for written informed consent was waived. Patients who underwent chest CT scans for suspected or known ILD between January 2015 and October 2024 were eligible. The inclusion criteria were: (1) the presence of parenchymal changes associated with ILD and (2) availability of HRCT and CCT reconstructions from the same CT scan in a lung kernel. The exclusion criteria were as follows: (1) the presence of any artifacts that could impact lung parenchyma quantification, (2) neoplastic lesions, (3) extensive pleural effusions, or (4) other significant non-parenchymal abnormalities that could skew quantitative analysis.

### CT protocol and analysis

Chest CT scans were acquired using several CT scanners with detector row configurations ranging from 64 to 256 rows. The scanners included the Siemens SOMATOM Perspective, Siemens SOMATOM Force, Siemens SOMATOM Definition AS+, Siemens SOMATOM Definition Flash, Siemens Sensation Cardiac 64 (all from Siemens Healthineers, Erlangen, Germany), and GE Revolution CT (GE Healthcare, Chicago, IL, USA). All exams were performed as volumetric scans and acquired during full inspiration in either supine or prone position, depending on individual patient needs. Imaging was conducted with or without the administration of intravenous CM based on clinical requirements. A detailed summary of scan parameters for each CT scanner is provided in Supplementary Table S1.

Visual analysis of the lung parenchyma was performed in HRCT lung kernel reconstruction in a lung window setting. ILD pattern was graded as either usual interstitial pneumonia (UIP) or non-UIP. The administration of CM was documented, noting whether the scans were contrast-enhanced or non-enhanced. Slice thickness and the number of axial images per reconstruction for HRCT and CCT were extracted from the DICOM data.

### Quantitative lung parenchyma analysis

**Fig. 1:**
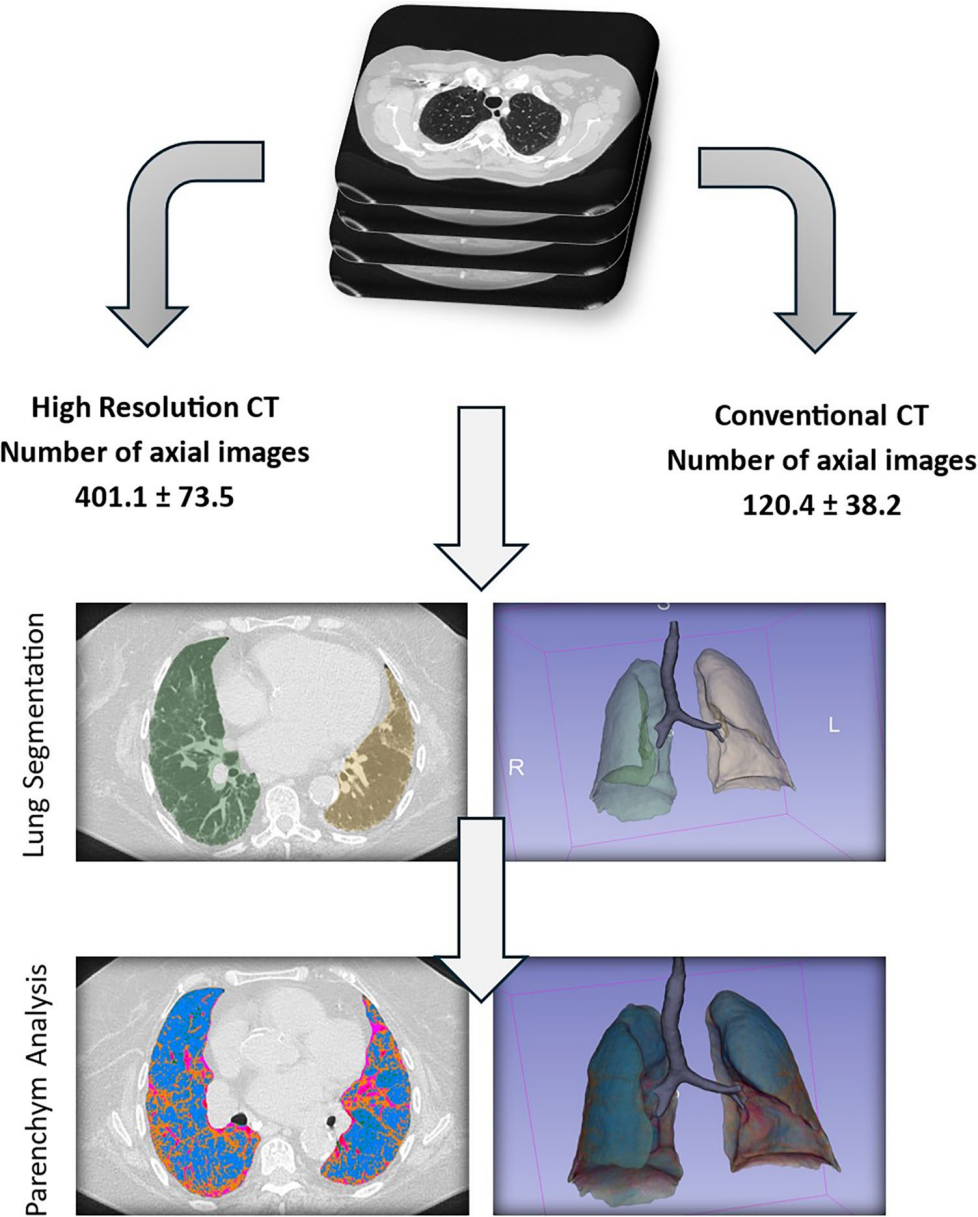
Workflow from DICOM data import to quantitative lung analysis using Lung CT Analyzer. A 3D volumetric lung mask is created separately for HRCT and CCT reconstructions (green: right lung; yellow: left lung). The masks undergo threshold-based quantitative analysis, resulting in a second lung mask with distinct color coding for each defined threshold component. DICOM = Digital Imaging and Communications in Medicine, HRCT = High-resolution computed tomography, CCT = Conventional computed tomography

DICOM data were imported into 3D Slicer (version 5.6.2). The analysis was conducted using the Lung CT Analyzer from the Chest Imaging Platform, separately for HRCT and CCT in lung kernel reconstruction (Fig. 1). The initial step involved creating a lung mask through semi-automated segmentation using the Lung CT Segmenter module. This required placing six markers on each lung and at least one marker to identify the airways or trachea, resulting in the creation of a 3D volumetric lung mask. In case of inadequate segmentation results, the process was repeated with the placement of additional markers in both lungs or airways.

**Fig. 2:**
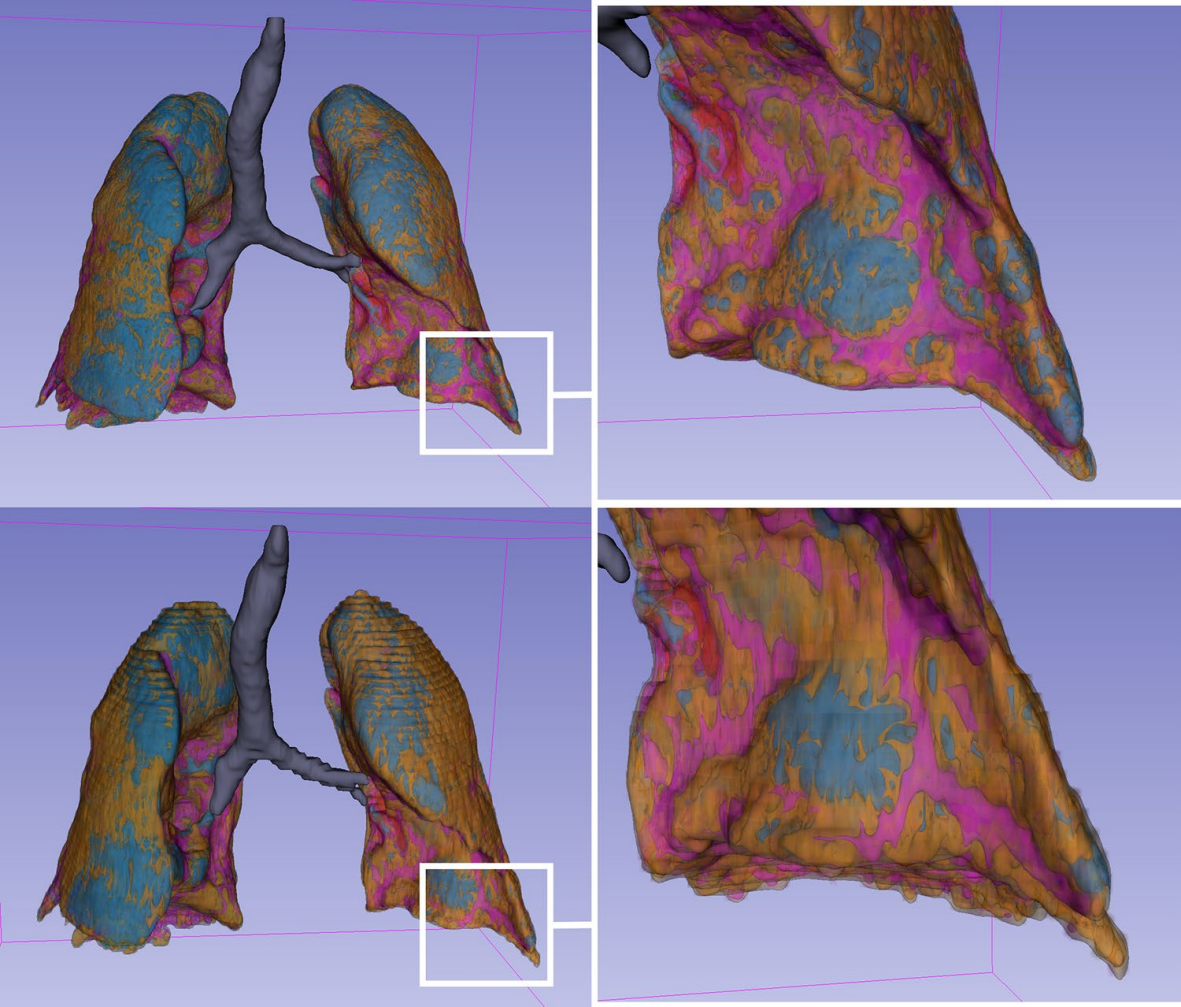
Lung mask creation using different slice thickness reconstructions. The upper row illustrates the 3D volumetric lung mask creation using HRCT reconstruction, while the lower row shows the process with CCT reconstruction. The difference in slice thickness is evident upon visual inspection: in the upper row (slice thickness 0.625 mm), the lung contours appear smooth, whereas in the lower row (slice thickness 3 mm), they appear ladder-like with visibly thicker sections. HRCT = High-resolution computed tomography, CCT = Conventional computed tomography

Once the lung mask was established, the Lung CT Analyzer module was used for quantitative analysis of the lung parenchyma (Fig. 2). The analysis was based on predefined Hounsfield unit (HU) thresholds to quantify total lung volumes and specific volumes of functional parenchyma, emphysematous areas, ground-glass opacities (GGO), consolidation, and affected lung parenchyma. Results were generated for the entire lung as well as separately for the right and left lungs. The HU thresholds were defined as follows: emphysema (− 1050 to − 950 HU), functional (− 950 to − 750), GGO (− 750 to − 400 HU), and consolidation (− 400 to 0 HU). Affected lung parenchyma was calculated as the sum of GGO and consolidation areas. Intrapulmonary vessels were subtracted from the final analysis to enhance the accuracy of parenchymal volume quantification.

### Clinical and laboratory data

Clinical and laboratory data were obtained retrospectively from the hospital’s information system. Demographic data (age at time of CT scan sex), smoking status, body mass index, family history of ILD, presence of selected chronic diseases (arterial hypertension, diabetes mellitus, coronary heart disease, dyslipidemia, chronic obstructive lung disease) were collected. Laboratory parameters included hemoglobin, creatinine, and estimated glomerular filtration rate. Lung function parameters, blood gas analysis results, and 6-min walking test data were also reviewed.

### Statistical analysis

Statistical analysis was performed using SPSS software (version 29.0, SPSS Inc., Chicago, IL, USA). A p-value of less than 0.05 was considered statistically significant. Data distribution was assessed visually using Q-Q plots and statistically with the Shapiro–Wilk test. Continuous variables are reported as mean ± standard deviation [SD] and range, while categorical variables are presented as frequencies and percentages. The paired samples t-test was used to compare results between HRCT and CCT for normally distributed data, whereas the Wilcoxon Signed-Rank test was applied when the assumption of normality was violated. To assess the magnitude of the differences, the effect size was calculated and expressed using Cohen's *d* for normally distributed data, where values of 0.2, 0.5, and 0.8 were interpreted as small, medium, and large effects, respectively [25]. For non-normally distributed data analyzed using the Wilcoxon Signed-Rank test, the effect size was calculated as: r = Z/√N, with values of 0.1, 0.3, and 0.5 interpreted as small, medium, and large effects [26]. Repeated measures ANOVA was conducted to evaluate the effects of the ILD pattern and contrast media application on the results, treating them as potential confounding factors.

## Results

### Study group characteristics

**Fig. 3:**
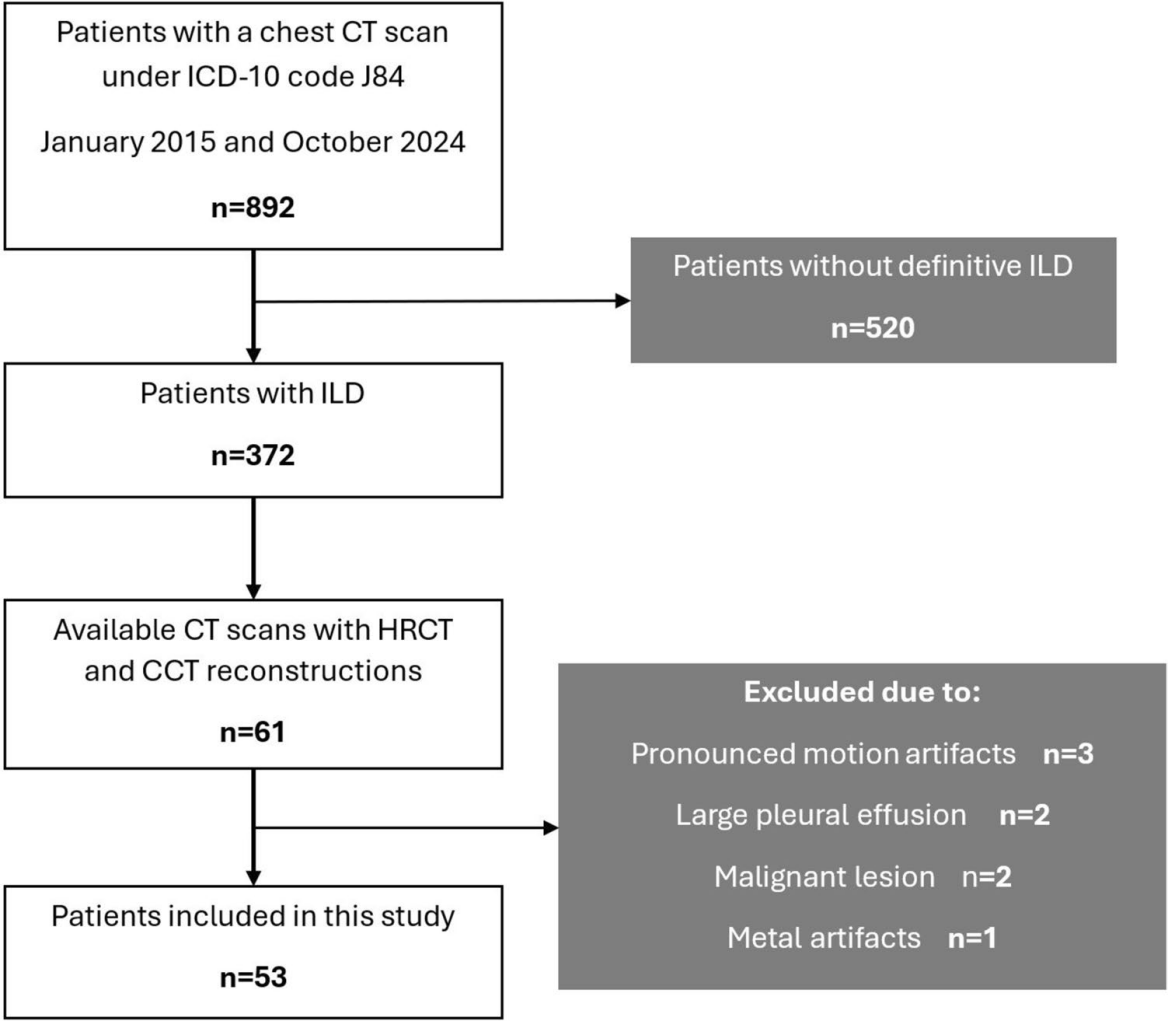
Flowchart of the study

A total of 53 patients were included in this study (Fig. 3) with a mean age of 64.3 ± 14.1 [SD] years (range 28–83 years), comprising 30 males (56.6%) and 23 females (43.4%). The most common causes of ILD were systemic sclerosis (n = 18, 34.0%), idiopathic pulmonary fibrosis (n = 11, 20.8%), interstitial pneumonia with autoimmune features (n = 7, 13.2%) and hypersensitive pneumonitis (n = 6, 11.3%) and. Complete patient’s characteristics are shown in Table 1.

**Table 1:** Patient characteristics of the study population

Characteristic	Total (n = 53)
Sex (Male:Female)	30:23
Age (years)	64.3 ± 14.1 (28–83)
BMI (kg/m^2^)	25.8 ± 5.0 (17.8–37.9)
Current or past smoker, No. (%)	22/53 (41.5)
Family history of ILD, No. (%)	2/53 (3.8)
Arterial hypertension, No. (%)	30/53 (56.6)
Diabetes mellitus, No. (%)	7/53 (13.2)
Coronary heart disease, No. (%)	22/53 (41.5)
Dyslipidamia, No. (%)	12/53 (22.6)
COPD, No. (%)	6/53 (11.3)
Hemoglobin (g/dl)	13.9 ± 3.9 (9.9–30.5)
eGFR (ml/min)	76.0 ± 21.5 (21.9–117.2)
NT-proBNP (pg/ml)	843.23 ± 1163.9 (48–4403)
VC, % predicted	78.7 ± 21.1 (36.5–123.0)
FVC, % predicted	77.0 ± 19.1 (35.8–116.0)
FEV1, % predicted	77.6 ± 18.4 (41.5–121.0)
FEV1/FVC	84.5 ± 13.4 (53.3–114.0)
TLC, % predicted	91.5 ± 23.7 (52.0–146.0)
DL_CO_, % predicted	59.5 ± 19.6 (20.0–105.0)

*ILD* interstitial lung disease, *COPD* chronic obstructive pulmonary disease, *eGFR* estimated glomerular filtration rate, *VC* vital capacity, *FVC* forced vital capacity, *FEV1* forced expiratory volume in first second, *TLC* total lung capacity, *DLCO* diffusing capacity for carbon monoxide

### CT analysis

The mean slice thickness of HRCT scans was 0.9 ± 0.2 mm [SD] (range 0.625–1.250 mm), with a mean number of axial images of 401.1 ± 73.5 [SD] (range 243–556). In comparison, the mean slice thickness of CCT scans was 3.2 ± 0.6 mm [SD] (range 2.5–5.0 mm), with a mean number of axial images of 120.4 ± 38.2 [SD] (range 61–245).

Based on visual assessment, a UIP pattern was identified in 17 patients (32.1%) and a non-UIP pattern in 36 patients (67.9%). Contrast-enhanced scans were performed in 31 patients (58.5%), while non-enhanced scans were performed in 22 patients (41.5%).

### Quantitative lung parenchyma analysis

**Fig. 4:**
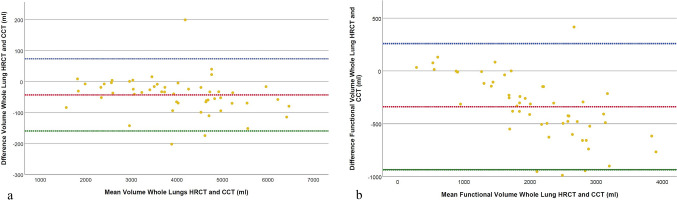
Comparison of overall lung volume (**a**) and functional lung volume (**b**) quantification results between HRCT and CCT reconstructions. The Bland–Altman plot illustrates the agreement between HRCT and CCT volume measurements. The red dotted line represents the mean difference between methods, the blue dotted line indicates the upper limit of agreement (mean + 1.96 × SD), and the green dotted line shows the lower limit of agreement (mean − 1.96 × SD). HRCT = High-resolution computed tomography, CCT = Conventional computed tomography, SD = Standard deviation

A statistically significant difference was observed in the overall estimated lung volumes between HRCT (3957.1 ± 1207.2 ml [SD], range 1536.7–6429.8 ml) and CCT (4000.6 ± 1323.4 ml [SD], range 1621.1–6510.0 ml, *p* < 0.001, *d* = − 0.733) (Table 2), with similar observations for the right and left lung (Table 3). Statistically significant differences were also detected in the functional lung volume for the whole lung, with HRCT measurements (1904 ± 743.5 ml [SD], range 303.9–3537.9 ml) being lower than those derived from CCT reconstructions (2243.4 ± 942.4 ml [SD], range 168.0–4296.5 ml, *p* < 0.001, *d* = 1.112) (Fig. 4). This was also detected for the right lung (Table 3).

**Table 2:** Results of the quantitative lung analysis for the whole segmented lung

Parenchyma component	HRCT volume (mL)	CCT volume (mL)	*P* value	Cohen's d
Lung volume overall	3957.1 ± 1207.2 (1536.7–6429.8)	4000.6 ± 1323.4 (1621.1–6510.0)	** < 0.001**	** − 0.733**
Functional parenchyma	1904 ± 743.5 (303.9–3537.9)	2243.4 ± 942.4 (271.0–4294.0)	** < 0.001**	**1.112**
Emphysema	766.9 ± 568.3 (10.0–2543.9)	482.6 ± 454.4 (0.4–1765.3)	** < 0.001**	− 0.819*
GGO	1013.9 ± 280.2 (384.9–1822.6)	1024.3 ± 327.0 (387.5–1867.0)	0.628	− 0.067
Consolidation	271.6 ± 128.4 (100.0–704.9)	252.0 ± 126.3 (100.3–692.6)	** < 0.001**	− 0.558*
Affected parenchyma	1285.5 ± 362.0 (497.7–2150.8)	1276.4 ± 413.5 (495.1–2218.3)	0.675	0.058

Bolded p-values indicate statistical significance, defined as *p* < 0.05

*Effect size (r) calculated using the Wilcoxon Signed-Rank test for non-normally distributed data. *HRCT* high resolution computed tomography, *CCT* conventional computed tomography, *GGO* ground glass opacity

**Table 3:** Results of the quantitative lung analysis for the left and right lung

Parameter	Right lung			Cohen's d	Left lung			Cohen's d
	HRCT volume (mL)	CCT volume (mL)	*P* value		HRCT volume (mL)	CCT volume (mL)	*P* value	
Lung volume overall	1286.0 ± 362.0 (810.5–3619.1)	1276.4 ± 413 (847.4–3679.1)	** < 0.001**	** − 0.675**	1795.6 ± 619.2 (514.7–3057.3)	1814.8 ± 623.3 (490.3–3049.0)	** < 0.001**	** − 0.635**
Functional parenchyma	1480.4 ± 649.8 (204.2–3058.1)	1510.5 ± 705.2 (117.5–3141.8)	**0.023**	**0.321**	1191.1 ± 596.7 (144.8–2496.1)	1213.7 ± 646.9 (86.8–2554.2)	0.070	0.254
Emphysema	431.4 ± 314.6 (5.4–1437.8)	270.2 ± 251.3 (0.4–999.8)	** < 0.001**	− 0.824^*^	337.3 ± 259.9 (4.6–1106.2)	228.0 ± 221.5 (0.1–814.4)	** < 0.001**	− 0.744^*^
GGO	540.7 ± 144.0 (202.8–984.3)	546.0 ± 174.9 (203.4–1018.3)	0.652	0.062	473.3 ± 146.3 (182.2–838.3)	478.3 ± 165.5 (184.0–848.7)	0.617	0.069
Consolidation	140.4 ± 60.5 (53.9–341.3)	129.3 ± 56.1 (55.8–306.0)	** < 0.001**	− 0.555^*^	131.2 ± 69.5 (46.1–363.6)	122.7 ± 72.0 (44.5–387.9)	**0.004**	− 0.520^*^
Affected parenchyma	681.1 ± 181.6 (264.1–1130.8)	675.3 ± 211.5 (262.9–1181.5)	0.625	** − **0.068	604.5 ± 190.4 (233.6–1034.3)	602.9 ± 213.1 (232.1–1133.4)	0.881	** − **0.021

Bolded p-values indicate statistical significance, defined as *p* < 0.05

*Effect size (r) calculated using the Wilcoxon Signed-Rank test for non-normally distributed data. *HRCT* high resolution computed tomography, *CCT* conventional computed tomography, *GGO* ground glass opacity

**Fig. 5:**
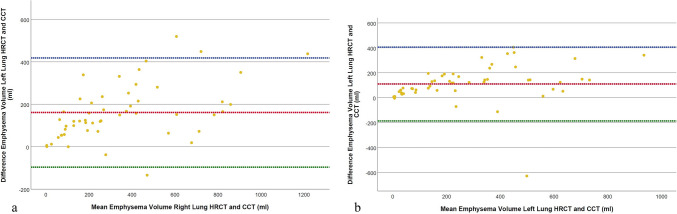
Comparison of emphysema volume quantification results between HRCT and CCT reconstructions for the right (**a**) and left lung (**b**). The Bland–Altman plot illustrates the agreement between HRCT and CCT volume measurements. The red dotted line represents the mean difference between methods, the blue dotted line indicates the upper limit of agreement (mean + 1.96 × SD), and the green dotted line shows the lower limit of agreement (mean − 1.96 × SD). HRCT = High-resolution computed tomography, CCT = Conventional computed tomography, SD = Standard deviation

The emphysema volume quantification from the whole lung showed a statistically significant difference, with CCT measurements (482.6 ± 454.4 ml [SD], range 0.4–1765.3 ml) being lower than those in HRCT (766.9 ± 568.3 ml [SD], range 10.0–2543.9 ml, *p* < 0.001, r = − 0.819). This was also found in separate analyses of the right and left lung, with significant differences noted for the right lung emphysema volume between HRCT (431.4 ± 314.6 ml [SD], range 5.4–1437.8 ml) and CCT (270.2 ± 251.3 ml [SD], range 0.4–999.8 ml, *p* < 0.001, r = − 0.824), as well as for the left lung between HRCT (337.3 ± 259.9 ml [SD], range 4.6–1106.2 ml) and CCT reconstructions (228.0 ± 221.5 ml [SD], range 0.1–814.4 ml, *p* < 0.001, r = − 0.744) (Fig. 5).

**Fig. 6:**
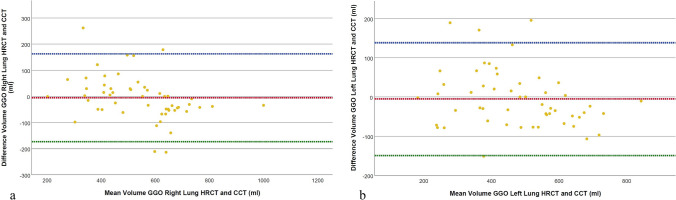
Comparison of ground-glass opacity (GGO) volume quantification results between HRCT and CCT reconstructions for the right (**a**) and left lung (**b**). The Bland–Altman plot illustrates the agreement between HRCT and CCT volume measurements. The red dotted line represents the mean difference between methods, the blue dotted line indicates the upper limit of agreement (mean + 1.96 × SD), and the green dotted line shows the lower limit of agreement (mean − 1.96 × SD). GGO = Ground-glass opacity, HRCT = High-resolution computed tomography, CCT = Conventional computed tomography, SD = Standard deviation

Furthermore, a statistically significant difference was noted in the consolidation volume of the whole lung between HRCT (271.6 ± 128.4 ml [SD], range 100.0–704.9 ml) and CCT (252.0 ± 126.3 ml [SD], range 100.3–692.6 ml, *p* < 0.001, r = − 0.558). This was also true for the right and left lung (Table 3). In contrast, the volume of GGO did not show statistically significant differences in measurements for the whole lung between HRCT (1013.9 ± 280.2 ml [SD], range 384.9–1822.6 ml) and CCT (1024.3 ± 327.0 ml [SD], range 387.5–1867.0 ml, *p* = 0.628, *d* = − 0.067). Similar results were observed in GGO quantification for the right and left lung (Fig. 6).

Overall, the volume of affected lung parenchyma did not show statistically significant differences between different slice reconstructions for the whole lung (*p* = 0.675, *d* = 0.058), the right lung (*p* = 0.569, *d* = -0.068), or the left lung (*p* = 0.696, *d* = -0.021). The complete results of the volumetric lung parenchyma quantification, along with their respective p-values, are presented in Table 2 for the whole lung and Table 3 for the right and left lungs.

### Impact of ILD pattern and CM application

There was no statistically significant interaction of contrast media application or ILD pattern on the results of the quantitative analysis for any component of the whole lung, as well as for the right and left lungs. The complete results are provided in Supplementary Tables S2, S3, and S4.

## Discussion

This study is the first to evaluate the effect of different slice thickness reconstructions on threshold-based quantification of ILD using the Lung CT Analyzer software. Our findings demonstrate that slice thickness plays a crucial role in the volumetric assessment of emphysema, consolidation areas, functional lung parenchyma, and overall lung volume estimation. In contrast, the volumes of GGO areas and the overall quantification of affected lung parenchyma appeared unaffected by variations in slice thickness. For most differences in volume quantification between slice thicknesses, the effect size was medium to large (Table 4). CM application and specific ILD patterns were identified as potential confounding factors; however, no significant interaction with our results was observed in any measured ILD component. Our findings indicate that variations in slice thickness heterogeneously affect CT volumetric quantification of ILD components, underscoring the importance of standardized protocols and the need for caution when interpreting quantitative results from CT scans with different slice thicknesses.

**Table 4:** Summary of slice thickness effects for each analyzed component

Parenchyma component	Volume difference CCT/HRCT	Effect size
Lung volume overall	Overestimated	Medium
Functional parenchyma	Overestimated	Small to large
Emphysema	Underestimated	Large
GGO	Unaffected	Negligible effect
Consolidation	Underestimated	Large
Affected parenchyma	Unaffected	Negligible effect

The differences in volumes are presented as a comparison of CCT to HRCT. The effect size is reported as Cohen's d for the paired t-test or as the effect size (r) from the Wilcoxon signed-rank test. *HRCT* high-resolution computed tomography, *CCT* conventional computed tomography, *GGO* ground glass opacity

Quantitative lung CT analysis has transitioned from a research tool to a widely accepted method for monitoring progression and assessing severity of ILD [27]. Threshold-based CT quantification of lung parenchyma enables differentiation between normal and fibrotic lung, as well as classification of parenchymal areas into categories such as functional lung, emphysema, GGO, or consolidation [28–30]. Previous studies have demonstrated a good correlation between the results of threshold-based lung quantification, visual CT assessments, and clinical parameters [31, 32]. The influence of several scan parameters and image reconstruction methods have been evaluated for quantitative lung analysis. The reconstruction kernel is of critical importance, as there is a significant influence of different reconstruction kernels on the results of quantitative lung analysis [33–35]. Although we included chest CT scans from different manufacturers, all reconstructions used for analysis were designed for evaluating lung parenchyma and fell into the category of sharp or high-frequency kernels [33, 36].

Several studies have investigated the effect of different scan parameters and reconstruction methods on the quantification of lung nodules, generally finding that most acquisition parameters have minimal impact on the results. However, slice thickness stands out as a key factor that significantly affects nodule volumetry, with thinner slices (≤ 0.75 mm) providing more accurate volume measurements compared to thicker slices (≥ 1.5 mm) [12]. Interestingly, subsolid or ground-glass nodules are less affected by slice thickness compared to higher-attenuation solid nodules, whereas reconstruction algorithms have a stronger effect on the quantification of ground-glass nodules than on solid, higher-attenuation nodules [15].

Regarding interstitial lung changes, previous studies have reported that an increase in slice thickness, as well as the use of sharp kernel reconstructions, leads to an increase in CT quantification of low-attenuation areas or emphysema volume compared to reconstructions with lower slice thickness and soft kernels [37, 38]. Another study demonstrated that the proportion of emphysema decreases as slice thickness increases and reconstruction kernel sharpness increases. This effect may be explained by the enhanced spatial resolution, which more precisely captures small low-attenuation areas while reducing pixel attenuation [18]. Similar to these studies, we observed an underestimation of emphysema volume in CCT reconstructions with higher slice thickness compared to HRCT reconstructions. The volume quantification of ground-glass areas was not affected by slice thickness, aligning with findings from previous studies on subsolid or ground-glass lung nodules, where slice thickness does not significantly impact the quantification of lower attenuation ground-glass nodules [15]. Higher attenuation areas or lung consolidations were strongly affected by slice thickness, which is consistent with findings from studies on solid lung nodules [15]. However, no comparable studies have specifically evaluated the impact of slice thickness on high-attenuation areas in ILD patients. Our results highlight this gap in literature and provide valuable insight on the interaction between slice thickness and high attenuation areas, especially since these areas are known risk factors for ILD mortality and their correct quantification could impact clinical decision making for this patient group [39, 40].

In a previous study conducted on patients with interstitial lung disease (ILD) associated with systemic sclerosis, a section thickness of 5 mm resulted in an underestimation of lung fibrosis extent compared to quantification performed using a 1 mm section thickness [24]. Although the impact of different slice thicknesses has been evaluated for the visual assessment of individual ILD components [41] and for quantitative lung analysis based on radiomic feature extraction [42], we did not identify any comparable studies that assessed the effect of slice thickness on the threshold-based quantification of individual parenchymal changes in ILD. An important observation in our study is that slice thickness does not affect the overall estimation of affected lung parenchyma in ILD, which contrasts with the mentioned previous study [24]. This discrepancy may reflect differences in lung quantification methods used, as well as variations in patient cohorts and disease patterns.

Previous studies reported that CM application affects the results of quantitative lung analysis by increasing overall lung density and therefore reducing the estimated volume of lung emphysema [43]. In our study, CM application showed no statistically significant interaction with the volume measurements of any ILD component [43]. Similarly, the ILD pattern, classified as UIP or non-UIP, did not show a statistically significant interaction with our results.

Besides the retrospective design of this study, several limitations must be acknowledged. Firstly, the relatively small sample size affects the overall statistical power of our study, limiting the ability to detect subtle differences between the two reconstruction thicknesses. Secondly, several CT scanners with varying detector configurations were used, and although all scans were standardized to lung reconstruction kernels, differences between scanners may have introduced variability in image quality and quantification accuracy. While lung reconstruction kernels were used uniformly, variations in kernel versions across scanner types could have impacted the results. Thirdly, the use of contrast media was also a potential limitation, as it was not systematically controlled across all patients.

In conclusion, our findings indicate that slice thickness has a significant impact on the threshold-based volumetric quantification of ILD. While certain analyzed components, such as GGO, remain stable across different slice thicknesses, others, such as emphysema and consolidation volumes, are highly sensitive to slice thickness variability. An important finding is that the estimation of overall affected lung parenchyma in patients with ILD is not influenced by slice thickness, which potentially allows for the quantitative comparison of chest CT scans with different slice thickness reconstructions. The ILD pattern and CM application did not affect the results of any analyzed ILD component, although these findings are limited by the sample size of this study. These findings underscore the importance of standardizing imaging protocols and highlight the need for caution when interpreting results from different CT protocols in both research and clinical settings, especially since our study setting reflects a more real-world scenario where exams are pooled from different CT scanners and protocols. Further research is needed to verify our results and to detect more subtle differences, especially regarding CM applications and the impact of different ILD patterns.

## Supplementary Information

Below is the link to the electronic supplementary material.

Supplementary file1 (PDF 198 KB)
