# Supplementary material for: Effect of slice thickness on quantitative analysis of interstitial lung disease: a retrospective volumetric chest CT study
Source: Radiol Med. 2025 May 27;130(8):1172–82. doi: 10.1007/s11547-025-02023-w (PMC12367816; doi:10.1007/s11547-025-02023-w)
Supplement: Supplementary file 1 — Supplementary file1 (PDF 198 KB) [file 11547_2025_2023_MOESM1_ESM.pdf]

Scan parameter	*GE Revolution*	*Sensation Cardiac 64*	*SOMATOM Force*	*SOMATOM Definition Flash*	*SOMATOM Perspective*	*SOMATOM Definition AS+*
Scan type	Helical	Helical	Helical	Helical	Helical	Helical
Direction	Craniocaudal	Craniocaudal	Craniocaudal	Craniocaudal	Craniocaudal	Craniocaudal
Inspiration/Expiration	Inspiration	Inspiration	Inspiration	Inspiration	Inspiration	Inspiration
Detector row	256	64	96	96	128	128
Kernel reconstruction	HD Lung	B70f	Bl64d\3	I70f\3	B70s	Br51f\2
Tube current	Modulated	Modulated	Modulated	Modulated	Modulated	Modulated
Tube voltage	100-255 kv	120 kv	90 – 110 kv	100 kv	110 – 130 kv	100 kv
Pitch	0.992	1.399	0.599	1.200	1.000	1.000
Gantry tilt	0	0	0	0	0	0
Collimation	80 mm	60 mm	60 mm	60 mm	60 mm	60 mm
Matrix	512 x 512	512 x 512	512 x 512	512 x 512	512 x 512	512 x 512

Supplementary Table 1. Scan parameters from each included CT scanner.

	CM application				***ILD pattern***			
Parameter	**F**	**df**	**p-value**	**partial η²**	**F**	**df**	**p-value**	**partial η²**
Lung volume	2.051	1, 49	0.158	0.047	0.047	1, 49	0.128	0.047
Functional parenchyma	0.388	1, 49	0.536	0.008	0.001	1, 49	0.996	0.001
Emphysema	0.016	1, 49	0.898	0.001	0.022	1, 49	0.882	0.001
GGO	0.185	1, 49	0.669	0.004	0.508	1, 49	0.479	0.010
Consolidation	0.055	1, 49	0.816	0.001	0.099	1, 49	0,755	0.002
Affected parenchyma	0.224	1, 49	0.638	0.005	0.590	1, 49	0.446	0.012

Supplementary Table 2. Results of the repeated-measures ANOVA for the whole lung, assessing the interactions of CM application and ILD pattern on quantitative lung parameters. CM = Contrast Media, ILD = Interstitial Lung Disease, GGO = Ground-Glass Opacity

	CM application				*ILD pattern*			
Parameter	**F**	**df**	**p-value**	**partial η²**	**F**	**df**	**p-value**	**partial η²**
Lung volume	2.146	1, 49	0.149	0.042	0.035	1, 49	0.200	0.033
Functional parenchyma	1.256	1, 49	0.268	0.025	0.005	1, 49	0.944	0.001
Emphysema	0.106	1, 49	0.746	0.002	0.009	1, 49	0.746	0.001
GGO	0.278	1, 49	0.601	0.006	0.207	1, 49	0.651	0.004
Consolidation	0.197	1, 49	0.659	0.004	0.004	1, 49	0.949	0.001
Affected parenchyma	0.389	1, 49	0.536	0.008	0.221	1, 49	0.641	0.004

Supplementary Table 3. Results of the repeated-measures ANOVA for the right lung, assessing the interactions of CM application and ILD pattern on quantitative lung parameters. CM = Contrast Media, ILD = Interstitial Lung Disease, GGO = Ground-Glass Opacity

	CM application				ILD pattern			
Parameter	**F**	**df**	**p-value**	**partial η^2**	**F**	**df**	**p-value**	**partial η^2**
Lung volume	1.076	1, 49	0.305	0.021	2.146	1, 49	0.149	0.042
Functional parenchyma	0.327	1, 49	0.570	0.007	0.174	0.678	0.004	0.009
Emphysema	1.563	1, 49	0.217	0.031	1.025	1, 49	0.316	0.031
GGO	0.087	1, 49	0.770	0.002	0.964	1, 49	0.331	0.019
Consolidation	0.001	1, 49	0.999	0.000	0.266	1,49	0.608	0.005
Affected parenchyma	0.222	1, 49	0.640	0.005	0.821	1, 49	0.369	0.016

Supplementary Table 4. Results of the repeated-measures ANOVA for the left lung, assessing the interactions of CM application and ILD pattern on quantitative lung parameters. CM = Contrast Media, ILD = Interstitial Lung Disease, GGO = Ground-Glass Opacity
